# Epidemiological findings on interventional cardiology procedures during the COVID-19 pandemic: A multi-center study

**DOI:** 10.1016/j.ihj.2021.06.016

**Published:** 2021-07-01

**Authors:** Stefano Albani, Hugo Vinhas, Georgina Fuertes Ferre, Sandeep Basavarajaiah, Sophia Khattak, Giorgos Tzanis, Margherita Pizzato, Marco Toselli, Arif A. Khokhar, Giuseppe Musumeci, Francesco Giannini

**Affiliations:** a Division of Cardiology, Azienda Ospedaliera Ordine Mauriziano di Torino, Turin, Italy; b Centro Hospitalar Universitário Do Algarve, Faro, Portugal Algarve Biomedical Center, Faro, Portugal; c Interventional Cardiology Unit, Miguel Servet University Hospital, Zaragoza, Spain; d Heartlands Hospital, University Hospitals Birmingham NHS Foundation Trust, Sutton Coldfield, Birmingham, UK; e Department of Interventional Cardiology, Henry Dunant Hospital Center, Athens, Greece; f Department of Clinical Sciences and Community Health, Università Degli Studi di Milano, Milan, Italy; g Interventional Cardiology Unit, GVM Care & Research Maria Cecilia Hospital, Cotignola, Ravenna, Italy

**Keywords:** Epidemiology, Interventional cardiology, COVID-19

## Abstract

**Background:**

The rates of in-hospital mortality following percutaneous interventional procedures (PIP) during the COVID-19 pandemic period compared to the non-pandemic period has not been reported so far.

**Methods:**

We retrospectively enrolled all consecutive patients admitted for PIP across five centers from February 2020 to May 2020.

**Results:**

A total of 4092 PIP were performed during the reference periods. The total number of procedures dropped from 2380 to 1712 (28.0% reduction). Overall in-hospital mortality increased from 1.1% in 2019, to 2.6% in 2020 (63% relative increase).

**Conclusion:**

During the COVID-19 pandemic, in-hospital all-cause mortality significantly increased in patients admitted for cardiological PIP.

## 1 Background

The COVID-19 pandemic has had a significant impact on healthcare institutions and cardiology departments worldwide.^1^ Specifically, a dramatic reduction in the number of elective and urgent percutaneous procedures being performed in the worst affected countries has been reported.^2^ Recent reports, including a STEMI cohort from China,^3^ have suggested that this reduction in interventional activity may be associated with elevated short and longer-term mortality rates.^4^

However, to date, no data exist on the overall rate of in-hospital mortality for patients undergoing percutaneous interventions during the pandemic period compared to the previous year.

## 2 Methods

All consecutive patients who underwent percutaneous interventions in five high-volume European hospitals (Maria Cecilia Hospital, Care & Research, Italy; Birmingham Heartlands Hospital, Birmingham United Kingdom; Henry Dunant Hospital Center, Athens, Greece; University Hospital Center of Algarve, Faro, Portugal; Miguel Servet University Hospital, Zaragoza, Spain) between February to May 2020 (pandemic cohort) and between February to May 2019 (non-pandemic cohort) were included.

Demographic, procedural and outcome data were collected for all patients and for the following procedures: diagnostic coronary angiography (CA), percutaneous coronary interventions (PCI), transcatheter aortic valve replacement (TAVR) and percutaneous mechanical circulatory support systems including Intra-aortic balloon pump and Impella (Abiomed, Danvers, MA). Patients who performed more than one procedure were considered only once, following a priority-model, based on the complexity of the procedure itself: the more complex the procedure, the higher the priority.

Primary endpoint was the difference in mortality between the pandemic cohort and the non-pandemic cohort. Secondary endpoint was the difference in procedural volumes between the pandemic cohort and the non-pandemic cohort.

Continuous variables are presented as mean ± standard deviation (SD). Categorical variables are expressed as proportions. They were compared between groups using Chi-squared test. A *p* value < 0.05 was considered to indicate statistical significance. The study received institutional review board approval and informed consent was obtained in accordance to each institution's local policy.

## 3 Results

A total of 4092 percutaneous procedures (2380 in 2019 and 1712 in 2020) were performed during the reference periods in the two consecutive years (Table 1).

**Table 1:** Characteristic of patients treated at Birmingham Heartlands Hospital, Birmingham United Kingdom; Henry Dunant Hospital Center, Athens, Greece; University Hospital Center of Algarve, Faro, Portugal; Miguel Servet University Hospital, Zaragoza, Spain from February to May in 2019 and in 2020 (CA: Coronary Angiograms, PCI: Percutaneous Coronary Interventions, TAVR: Transcatheter Aortic Valves Replacement).

	Total Procedures					*p* value
	2019		2020		% change 2020 vs 2019	
	N	%	N	%		
	2336	57.7	1712	42.3		
**Procedure**						<0.01
CA	920	39.4	586	34.2	−36.3	
PCI	1349	57.7	1057	61.7	−21.6	
*CA/PCI ratio*	*0.68*		*0.56*			
TAVR	31	1.3	22	1.3	−29.0	
Impella and Intra-aortic balloon pump	15	0.6	1	0.1	−93.3	
Other procedures	21	0.9	46	2.7	119.0	
**Mortality**						<0.01
CA	4	0.2	7	0.4	75.0	
PCI	19	0.8	29	1.7	52.6	
TAVI	0	0.0	3	0.2	N/A	
Impella and Intra-aortic balloon pump	4	0.2	1	0.1	−75.0	
Other procedures	0	0.0	4	0.2	N/A	
**Month**					.	<0.01
February	532	22.8	523	30.5	−1.7	
March	591	25.3	437	25.5	−26.1	
April	580	24.8	310	18.1	−46.6	
May	633	27.1	442	25.8	−30.2	
**Status**						<0.01
In-hospital mortality	27	1.2	44	2.6	63.0	
**Covid-19**						
Negative			553	32.3		
Positive - *Tested before the procedure*			4	0.2		
Positive - *Tested after the procedure*			9	0.5		
Not tested/Unknown			1146	66.9		

During the COVID-19 pandemic the majority of patients were males (973, 56.9%) with a mean age of 66 (±12) years. A 28% reduction (rom 2380 to 1712) of overall procedures was noticed compared to the previous year. The number of CA, PCI, TAVI and the use of percutaneous mechanical circulatory support systems decreased by 36.3%, 21.6%, 29% and 93.3% between the two cohorts.

Among the coronary procedures performed, there was a significant change in the proportion of diagnostic-only versus percutaneous interventions performed between the two cohorts. There was a significant change in the relative proportions of CAs and PCIs between the two cohorts: CAs decreased from 39.9% in 2019 to 34.2% in 2020 (p < 0.01), while PCIs increased from 57.7% to 61.7% (p < 0.01) (Table 1). The CA/PCI ratio fell from 0.68 to 0.55.

Overall in-hospital mortality was 2.6% in 2020 (44 out 1712) and 1.1% (27 out 2380) in 2019 with an increase of the total in-hospital mortality of 63.0%.

## 4 Discussion

The main findings of the present study are the following:

1) There was a 28% decrease in the number of percutaneous interventional procedures performed during the COVID-19 pandemic.
2) The proportion of PCI to diagnostic only procedures being performed was higher during the pandemic with a CA/PCI ratio close to 0.5.
3) The overall in-hospital mortality for patients undergoing percutaneous interventions was doubled during the pandemic compared to the non-pandemic period with a relative increase of 63%.

The COVID-19 pandemic has resulted in a significantly elevated overall and cardiovascular mortality^5^^,^^6^ in Europe^7^ and in the United States.^8^

To our knowledge this is the first report demonstrating the elevated in-hospital mortality of patients undergoing interventional cardiology procedures, during the COVID-19 pandemic.

Prior studies have highlighted the increased mortality observed in COVID-19 patients with pre-existing cardiovascular disease, such as acute myocardial injury or previous coronary artery disease.

9, 10. A recent Chinese report of STEMI patients, revealed a 20 min time delay to achieve definitive revascularization, increased use of thrombolytic therapy and this translated into an elevated in-hospital mortality rate for these patients.^3^ These findings highlight the potential impact a pandemic could have on overall healthcare pathways, especially the STEMI network, which is considered a well-established time-critical pathway^11^, ^12^. Time delay also has a negative impact upon the management and outcomes of NSTEMI patients.^13^ All this works suggest that ACS hospital presentation delay due to the risk of nosocomial source of infection could certainly have had an impact on the outcome.

In addition, to a decrease in procedural volume, our study also highlights changes in attitudes and working practices. The reduction in the CA/PCI ratio from 0.68 to 0.55 may reflect efforts to select a more urgent patients who require revascularization. Furthermore, the impact of reduced bed-space and available staffing for non-COVID patients, coupled to a systemic decline in elective procedures, is a further factor to be considered.^14^ The lower number of mechanical support assisted PCIs, which is much increasing in number in these years,^15^ could be seen as a consequence of a probable reduction of elective activity.

Our findings should be interpreted considering the limits of retrospective data collection. These results are derived from five high volume European centers, which experienced their peak pandemic at different time periods in 2020. Unfortunately, indications for 2019 procedures were not available.

## 5 Conclusions

The in-hospital all-cause mortality for patients who underwent interventional cardiology procedures during the COVID-19 pandemic was doubled compared to the non-pandemic period.

Further studies should evaluate the reasons underlying the observed elevated in-hospital mortality to better inform and prepare healthcare and government policies in case of a relapse of the pandemic.

The authors report no financial relationships or conflicts of interest regarding the content herein.
